# Caring and Health of Close Family Members of Frail Older Persons Recently Discharged from Acute Hospital Care: A Comparative Cross-Sectional Study

**DOI:** 10.3390/nursrep14020069

**Published:** 2024-04-10

**Authors:** Christina Bökberg, Tove Lindhardt, Eva Björkman, Gerd Ahlström

**Affiliations:** 1 Department of Health Sciences, Faculty of Medicine, Lund University, 221 00 Lund, Sweden; christina.bokberg@med.lu.se; 2 Research Unit for Clinical Nursing, Department of Internal Medicine, Copenhagen University Hospital Herlev and Gentofte, Herlev Ringvej 75, 2730 Herlev, Denmark; tove.lindhardt@gmail.com; 3 Department of Care Science, Faculty of Health and Society, Malmö University, 205 06 Malmö, Sweden; eva.bjorkman@mau.se

**Keywords:** mental health, general health, acute hospital care, home care, multimorbidity, older persons, family caregiver, informal caregiver

## Abstract

Multimorbidity in older people is strongly linked to the need for acute hospital care, and caregiving activities usually become more complex after patients are discharged from hospital. This may negatively impact the health of close family members, although this has not been comprehensively investigated. This study aimed to explore the general and mental health of close family members caring for frail older (>65) persons recently discharged from acute hospital care, making assessments in terms of gender, relationship to the older person, and aspects of caring. A comparative cross-sectional study was conducted involving 360 close family members caring for frail older persons recently discharged from hospital. The statistical analyses included subgroup comparisons and associations to caring were examined. Half of the family members reported that their general and mental health was poor, with spouses reporting the poorest health. Female participants had significantly more severe anxiety, while males had significantly more severe depression. Providing care for more than six hours per week was associated with poor general health (OR 2.31) and depression (OR 2.59). Feelings of powerless were associated with poor general health (OR 2.63), anxiety (6.95), and depression (3.29). This knowledge may provide healthcare professionals with better tools in order to individualise support, preventing family members from exceeding their resources during these demanding periods.

## 1. Introduction

Caregiving by close family members is likely to become increasingly important in the future throughout Europe due to demographic changes, healthcare advances, the prioritisation of the policy “ageing in place”, and cost containments [1,2]. Given the ageing demographic trends and the increasing demand for care at home in most countries in the European Union (EU), a strategy has been developed for the creation of a carer-friendly society, where informal carers across Europe are recognised, empowered, and supported [2,3,4]. The amount of care and support provided by close family members to relatives is hard to calculate, but approximately 13% of persons above 50 years of age in OECD countries [5], and one in five adults in Sweden, provide care and support for a relative [6]. Although being a close family carer may seem voluntary, the extent and form of caring are rarely conscious choices [7,8].

Previous research has described the existence of conflicting feelings among close family members with regards to caring for a relative. On the one hand, close family members want to be involved and provide care and services based on their love of and responsibility for their relative; on the other hand, they can feel obligated due to remorse or a guilty conscience [9,10]. Often, they experience a loss of freedom due to the always-present feeling of responsibility, as well as feelings of guilt, irritation, and frustration [11], which may affect their own general and mental health. The consequences of these issues for close family members’ health have been widely described and summarised in systematic reviews. The results of these reviews showed the negative impact of caregiving for older persons on both the mental and physical health of close family members [12,13]. Sex (i.e., female), relationship (i.e., married), and a higher number of hours of provided care were found to have negative impacts on health [12]. Anxiety and depression are two major mental health problems that close family members might be burdened with when caring for an older person, and are sometimes designated in the literature by the term “caregiver burden” [13,14,15]. Depression and anxiety are associated with other health problems, such as fatigue and sleep disturbances [16], as well as higher rates of morbidity and mortality [17]. However, studies that compare groups of close family members caring for frail older persons and address the significance of gender, as well as both general and mental health, are rare [12,18].

Longevity is strongly associated with a high risk of frailty through multimorbidity, i.e., the presence of two or more chronic health conditions in an individual [19]. Multimorbidity is associated with functional impairment and care dependency, and it is strongly linked to the need for acute hospital care, increased primary healthcare, reduced continuity in the chain of care, and premature death [19,20,21,22]. In addition, the need for caregiving activities increases over time, and the needs typically become more complex after the older patient is discharged from acute hospital care [23,24,25,26]. Nurses in hospital settings have an important role to play in supporting family caregivers’ feelings of readiness for new and expanded caregiving roles after discharge [27]. Nurses need to be knowledgeable about the patient’s care pathway in order to identify barriers relevant to the family member’s care, such as a lack of relevant knowledge, a lack of trust and social support, and a lack of resources on the part of the patient and the family member, which may hinder successful communication between nurses and the family [28]. The demanding period after discharge from acute hospital care, often defined by an increased burden of care, may have a negative impact on the wellbeing and health of close family members. However, there is a knowledge gap in the literature regarding close family members’ general and mental health after a frail older relative is discharged from acute hospital care [29,30]. At this stage of the care pathway, attaining sufficient knowledge about family members’ self-reported health and the care needs of their close relative could provide nurses with information about whether a support programme should be developed.

### Aim

The aim of this study was to explore the general and mental health of close family members caring for frail older persons (≥65 years) recently discharged from acute hospital care, making assessments in terms of gender, relationship to the older person, and aspects of caring.

## 2. Materials and Methods

### 2.1. Design

This was a comparative cross-sectional study involving the close family members of older persons recently discharged from acute hospital care. Cross-sectional designs are often applied to measure the occurrence of health outcomes and explore the determinants of health within a specific population. This design does not allow for causal inferences, but instead provides information relevant to hypotheses and further research [31].

### 2.2. Sample

The sample consisted of the close family members of frail persons with multimorbidity, aged 65 years or older, who had recently been discharged from acute hospital care. A further criterion for inclusion was that the family member was the one providing the most informal care and support for the older person before hospitalisation. Family members who did not understand or speak Swedish were excluded. Family members were recruited consecutively from 13 acute medical wards at five hospitals across the south of Sweden, two of which were university hospitals and three of which were local hospitals, covering both urban and rural areas.

The recruitment of family members was initiated by asking the patients who met the inclusion criteria if they would like to supply the names of their family members and give permission for the researcher to contact their family members. After the patient was discharged from hospital, 426 eligible family members that had been named were contacted by phone by the researcher, who informed them about the study and asked if they were interested in participating. A total of 32 did not want to participate, and the remaining 394 received a questionnaire by post. Twenty-two declined to continue participation after receiving a reminder by phone. The reasons given were the burden of providing care, fatigue, grief, and emotional exhaustion in relation to the recent death of the older person. In a couple of cases, the family member was not sufficiently involved in the older person’s care after hospitalisation. Overall, 372 family members completed and returned the questionnaire, giving a response rate of 87.3%. After conducting quality control of the questionnaires, twelve family members were excluded due to an internal dropout of more than ten items.

The characteristics of the study group (*n* = 360) are shown in Table 1. The mean age was 63 years (SD 11.5), the median age was 62 years (range of 20–95 years), and most of the participants were female (64%). The female family members were most often adult children (61%; 141/229), followed by spouses (30%; 69/229). The men in the study were similarly adult children (57%; 75/131) and spouses (31%; 41/131). In total, one-third (34%) of participants were cohabiting with the older patient.

### 2.3. Measures

Mental health, specifically anxiety and depression, was measured using the self-reported hospital anxiety and depression scale (HAD) [32]. The questionnaire consists of 14 items that are equally distributed between two subscales, anxiety (HAD-A) and depression (HAD-D). A four-point Likert scale, ranging from 0 (not present) to 3 (considerable), constitutes the response alternatives. Thus, the subscale scores range from 0 to 21, where 0–7 points indicate no anxiety or depression, 8–10 points indicate mild anxiety or depression, and 11 or more points indicate severe anxiety or depression. The instrument has been translated and tested in different languages, including Swedish [33], and has proven to be reliable in terms of Cronbach’s alpha: α 0.84 for HAD-A and α 0.90 for HAD-D. The Cronbach’s alpha in this study was α 0.89 for HAD-A and α 0.88 for HAD-D.

Three questions from the recurring national survey “Family members providing care for close relatives—scope and consequences”, developed by the National Board of Health and Welfare [34], were included. One question concerned the family members’ assessment of their general health, and two questions assessed whether family members received support for their roles. The response alternatives are shown in Supplementary File S1. Permission to use the questions was obtained from the National Board of Health and Welfare.

Items regarding demographic characteristics, as well as those querying the number of caring hours and caring activities, were selected from the Swedish version of the Family Collaboration Scale (FCS) [35]. Furthermore, care-related items concerning responsibility for the older person’s wellbeing, sufficient formal care assistance, feelings of powerlessness, feelings of guilt, and feelings of not doing enough for the older person were included. The response alternatives for the survey are shown in Supplementary File S1. The Swedish version was evaluated and found to have psychometric properties equivalent to the revised Danish FCS [36].

### 2.4. Data Collection

A registered nurse stationed on each acute medical ward at the five hospitals was given information and written material about the study at a meeting with one of the researchers (EB). The contact nurse, in turn, gave easy-to-read information on an A3 sheet with enlarged typescript to the patient, who was given the opportunity to ask questions about the study. The patients were asked if they wanted their family members to be included in the study. If they did, the contact nurses handed the family members’ contact information in the form of telephone numbers to the researcher. The researcher then contacted the family members by telephone to give oral information about the study; at the same time, the researcher asked for permission to send the questionnaire, written information, a written consent form, and a prepaid return envelope. The questionnaire was completed within a month of their relative’s discharge from acute hospital care.

### 2.5. Statistics

In addition to obtaining descriptive statistics, subgroup comparisons and associations were examined. The Kruskal–Wallis test was used to compare the three subgroups of family members. Then, the Mann–Whitney U-test was applied to compare males and females, and this was also used to conduct a post hoc analysis when the results revealed significant differences. Fisher’s exact test was applied when the expected value was less than 5.

In order to identify associations between caring-related aspects and general and mental health, logistic regression analyses were performed. More than half of the family members reported “Not currently” in response to the two variables “Receive enough support from the health and social services” and “Getting enough help as a supportive person”, and these participants were excluded from the logistic regression analyses. Thus, the model contained seven independent variables, with adjustment for gender and the relationship to the older person. The dichotomisation of the variables used for the logistic regression analyses is described in Supplementary File S1.

A two-tailed *p*-value ≤ 0.05 was considered statistically significant. Analyses were performed in dialogue with a statistician and by using IBM SPSS Statistics version 26.

## 3. Results

### 3.1. General and Mental Health of Close Family Members

As shown in Table 2, there were no significant differences in terms of general health between men and women; however, regarding mental health, significantly more females reported severe anxiety (*p* = 0.018), and more males reported depression (*p* = 0.033). Half of the spouses reported their general and mental health to be poor, with mild or severe anxiety and depression (Table 2). Furthermore, the general health of the spouses was significantly poorer than that of the adult children and other family members (Mann–Whitney U-test *p* < 0.001).

The results regarding aspects of caring and gender (Table 3) revealed that females more often felt powerless (*p* = 0.007) and guilty (*p* = 0.034) concerning older relatives than males did. Males reported that they were receiving enough support from the relevant health and social services more often than females did (*p* = 0.020).

Significant differences were found between the subgroups of family members (Table 3). Spouses provided a greater number of hours of care per week than both adult children and other family members did (both *p* < 0.001), but adult children provided a greater number of hours of care than other family members did (*p* = 0.048). Spouses also performed a greater number of caring activities than both adult children and other family members did (*p* < 0.001 and 0.001).

Spouses felt more responsible for the older person receiving sufficient formal care assistance than adult children did (*p* = 0.022), but adult children felt more responsible than other family members did (*p* < 0.001). Both spouses and adult children felt more powerless than other family members did (*p* = 0.003 and 0.007, respectively). Adult children felt more guilty (*p* = 0.013) than other family members did, but no significant differences were found for spouses. Regarding support for the older person and themselves, at least half of all groups of family members answered “Not currently” on the items “Receive enough support from the health and social services” (49–58%) and “Receive enough help as a supportive person” (55–60%).

### 3.2. The Impact of Caring

The logistic regression analysis between aspects of caring and health (Table 4) showed that persons caring for more than six hours per week were twice as likely to have poor general health (OR 2.31 (CI 1.31–4.08)) and depression (2.59 (CI 1.46–4.62)). Furthermore, feeling powerless was more likely to be associated with poor general health (OR 2.63 (CI 1.39–4.97)), anxiety (6.95 (CI 37.72–12.99)), and depression (3.29 (CI 1.80–6.02)). In contrast, family members who felt responsible for the older person receiving sufficient formal care assistance were less likely to have poor general health themselves (OR 0.16 (0.38–0.69)) (Table 4).

## 4. Discussion

The main results revealed that close family members were negatively affected by caregiving, with half reporting anxiety and depression after their relative was discharged from acute hospital care. Devoting many hours while feeling powerless was related to poorer general and mental health. Spouses were the most vulnerable family members, had the poorest general and mental health, provided the greatest number of hours of care per week, and performed the highest number of care activities. The gender of the family member influenced mental health: women reported more severe anxiety, more frequent feelings of powerlessness, and more guilt than men did. Men were more depressed, even though they reported that, more often than not, they received enough support from health and social services.

Our results were in accordance with a review by Bom and colleagues, who investigated the effects on the health of subgroups of carers when providing informal, unpaid care for older people [12]. They found that females, spouses, and those providing many hours of care experienced adverse health effects, including depression and poor mental health [12]. It is evident from the literature that carers are most commonly women, and that they are involved in more and varied caring activities; men are more likely to provide practical help [1,2,37]. Many studies have found that female caregivers report poorer mental health (e.g., depression) than male caregivers, but this finding is not consistent across the literature [38,39,40,41]. To explain why more men than women reported depression in this study, further investigation is required. However, most studies include family carers as a homogeneous group or focus on patients with specific diagnoses. Therefore, further research needs to identify particularly stressful situations for close family members, including determining the amount and intensity of tasks required to cause stress. In our study, spouses were the most vulnerable family members, providing the greatest number of hours of care per week and performing the greatest number of care activities, while also having the poorest general and mental health. Aged spouses may have their own health problems and decreased energy and physical resources due to ageing, which might explain the poor general health seen in this group. Two scoping reviews found that spouse carers are mostly about the same age as their partner and usually cohabit with them. They are heavily involved in caring for partners with multiple needs and view their caring as a natural extension of their role as wife or husband [18,42]. There is extensive evidence of the negative impact of this practice, especially of long-term caring, on carers’ health. A consequence of increased longevity is the growing volume of older spouse carers, but these carers are often invisible in policy, research, and support programmes [18,42].

Home care is seen as a cost-effective way to avoid institutionalisation and enable older persons to remain living at home [2,43]. Despite the cost-effective benefits for society, there are negative consequences for close family members in terms of physical and mental health, such as psychological distress, financial and social strain, impaired family relationships, and a sense of hopelessness [11,40,41,44]. Despite this, 80% of all long-term care in Europe is provided by informal carers [2,43].

For older people, admission to acute hospital care is often associated with an ongoing deterioration of health, which means that an increased burden of care has been provided by the family member, possibly for some time, before hospital admission. Further, functional decline in older people has been widely described after hospitalisation [23,25,26], meaning that close family members will face even more and increasingly complex demands after the older person is discharged. Efforts have been made to develop algorithms in order to guide the type of care and level of care that best matches patients’ needs after discharge from acute hospital care [45]; however, the needs of close family members have not been included in those efforts.

More than half of the close family members in our study reported feelings of guilt and powerlessness, which was in line with the findings of a study conducted on a Danish sample of close family members [36]. Such feelings have been interpreted in terms of worry and a sense of insufficiency when resuming care responsibility after the hospitalisation of the older person [46]. The period before moving to a nursing home involves a process that could negatively affect close family members’ wellbeing, with the spouse experiencing loneliness, separation, grief, and exhaustion [47,48]. Adult children can experience constant worrying and a sense of guilt, and can come to distrust formal care [47,49,50]. This is in line with our results concerning anxiety, depression, and a sense of powerlessness. These results indicate that nurses need to have particular knowledge and competencies in order to identify signs of the deterioration of the mental wellbeing of close relatives when an older person has been discharged from acute hospital care. Nurses must also have a clear plan in order to support close family members in handling the often-increased caring responsibilities after discharge from acute hospital care, doing everything possible to prevent the further deterioration of health and exhaustion [27]. An initiative to address this was taken in a randomised controlled trial, in which 62 caregivers received an intervention programme to prepare them to care for older people after discharge from hospital. The intervention consisted of an explanation of the discharge letter, an assessment of caregiver support needs, an explanation of the prioritisation of urgent needs, and collaborative guidance provided by a specially trained nurse. The intervention group showed significantly improved readiness for new and extended care after discharge compared with the control group. This is a promising support programme, but it requires further testing before implementation in clinical practice [51].

As informal care is a cornerstone of long-term care in all European countries, it is no surprise that there are several intervention studies focusing on older people [2]. However, family carers who contribute to the success or failure of the patient’s care after discharge from hospital are only seen as a resource, not as individuals who need support themselves [51]. Empowering close family members, identifying their support needs, and easing their care burden are necessary tasks for nurses [51]. One reason for these tasks being insufficiently performed may be a lack of knowledge and experience of their application in clinical practice. Another reason is unclear legislation and the different functions and responsibilities of healthcare providers. In Sweden, support for family carers relates to social services legislation and is provided by social workers [7,8], while healthcare interventions are based on healthcare legislation and are mainly provided by registered nurses. Adopting a family approach, rather than solely relying on a patient-focused approach, could enable nurses to support family carers more effectively and holistically [52].

Some methodological aspects of this cross-sectional study need to be considered. Initial contact was made with older persons in the acute medical ward, who were asked permission to contact their family member. People with severe dementia or who were not conscious could not be asked if they wished for their close family member to be contacted about participation in the study. This reduced the possibility of generalising the results to family members caring for older people with dementia and serious cognitive impairment. As the study was conducted in Sweden and has a Nordic and European perspective, the results do not reflect caregiving by family members in low-income countries and in cultural contexts with great differences to European countries. Therefore, this study does not take full account of the complexity of the whole population of family carers [18].

The contact nurses working at the 13 medical wards were informed that they should only include close family members caring for older persons with multimorbidity, i.e., at least two diseases. However, the ethics committee decided that the older persons’ diagnoses should not be included in the questionnaire. This limited the generalisability of the results, since different diagnoses and the number of diagnoses could have impacted the burden placed on the family members, and therefore affected their general and mental health. The statistical associations observed were valid for the month following the discharge of patients with multimorbidity from acute hospital care. The question of whether these associations persist over time needs to be investigated in longitudinal studies before conclusions can be drawn about persistent poor health among family members.

Another weakness in this study was the unknown attrition rate. The contact nurses were not consistent in filling out the list regarding how many eligible older people did not consent to their family member’s participation. We estimated that factors such as a heavy workload and sick leave meant that not all of the older persons who met the inclusion criteria were asked about participation; therefore, there was no chance of their family members being included in the study. Furthermore, in the next step, the rate of non-consent to participation by the family member was 13.7% (54 of 426 eligible family members). The reasons given indicated an overwhelming emotional strain, which could imply that the family members included in the study had better general and mental health than those who dropped out. In order to obtain a representative sample, the researcher repeatedly telephoned each contact nurse at the five hospitals with reminders during the enrolment period, and also sent reminders to the family members who accepted the invitation. These reminders reduced the rate of non-response to a significantly lower level than would have been the case otherwise.

The HAD questionnaire has been psychometrically tested in several languages [33]. This is in contrast to the questions about support and care, but these have been found to be acceptable and comprehensible in practice [34,35,36]. However, this is a weakness that must be considered when interpreting the results.

## 5. Conclusions

This study contributes to closing the knowledge gap regarding the general and mental health of close family members after their older relative is discharged from acute hospital care. It was revealed that half of the spouses perceived their general and mental health to be poor, with mild or severe anxiety and depression, and that the general health of the spouses was significantly poorer than that of adult children and other family members. The knowledge from this study will enhance nurses’ awareness, in both acute hospital care and home care, of close family members needs for targeted individualised support, thereby preventing family members from exceeding their resources in this demanding period. This support has the potential for reducing the negative effects of caregiving on the general and mental health of close family caregivers.

## Figures and Tables

**Table 1:** Background data of the family members who participated in the study.

Background Variable	Total (n = 360)	Female (n = 229)	Male (n = 131)	Spouse (n = 110)	Adult Child (n = 215)	Other Family Member (n = 35)
	n (%)	n (%)	n (%)	n (%)	n (%)	n (%)
Age (n = 359)						
18–49	43 (12)	36 (16)	7 (5)	0 (0)	36 (17)	7 (20)
50–64	164 (46)	102 (45)	62 (47)	14 (13)	145 (67)	6 (17)
65–79	127 (35)	81 (36)	46 (35)	77 (70)	34 (16)	16 (46)
80+	25 (7)	9 (4)	16 (12)	19 (17)	0 (0)	6 (17)
Gender						
Female	229 (64)			69 (63)	141 (65)	20 (57)
Male	131 (36)			41 (37)	75 (35)	15 (43)
Cohabitating with the older person	124 (34)	75 (33)	49 (37)	110 (100)	10 (5)	4 (11)
School education						
Compulsory school	120 (33)	77 (34)	43 (33)	58 (53)	46 (21)	16 (46)
Gymnasium	126 (35)	68 (30)	58 (44)	26 (24)	94 (44)	7 (20)
University degree	114 (32)	84 (36)	30 (23)	26 (24)	76 (35)	12 (34)
Working	185 (51)	121 (53)	64 (49)	21 (19)	159 (74)	5 (14)

**Table 2:** General health and mental health, measured by HAD, with reference to gender and relationship.

Background Variable	Total		Female		Male			Spouse		Adult Child		Other Family Member		
	n (%)	Median (Q1–Q3)	n (%)	Median (Q1–Q3)	n (%)	Median (Q1–Q3)	*p* ^1^	n (%)	Median (Q1–Q3)	n (%)	Median (Q1–Q3)	n (%)	Median (Q1–Q3)	*p* ^2^
General Health		2 (2–3)		2 (2–3)		2 (2–3)	0.124		2.5 (2–3)		2 (2–3)		2 (1–2)	**0.001**
Good (1–2 p)	231 (64)		141 (62)		55 (50)			55 (50)		149 (69)		27 (77)		
Poor (3–5 p)	129 (36)		88 (38)		55 (50)			55 (50)		66 (31)		8 (23)		
Anxiety		7 (3–10)		7 (3–11)		6 (2–10)	**0.018**		7 (3–10)		6 (3–11)		2.5 (0–10)	0.372
No (0–6 p)	178 (50)		105 (46)		50 (46)			50 (46)		109 (51)		19 (56)		
Mild (7–10 p)	91 (26)		59 (26)		36 (33)			36 (33)		47 (22)		8 (23)		
Severe (≥11 p)	89 (24)		65 (28)		23 (21)			23 (21)		59 (27)		7 (21)		
Depression		5 (2–8)		6 (3–9)		5 (2–7)	**0.033**		7 (4–9)		5 (2–8)		4 (0–7)	0.060
No (0–6 p)	210 (58)		124 (54)		53 (48)			53 (48)		132 (61)		25 (71)		
Mild (7–10 p)	103 (29)		73 (32)		40 (36)			40 (36)		55 (26)		8 (23)		
Severe (≥11 p)	47 (13)		32 (14)		17 (16)			17 (16)		28 (13)		2 (6)		

^1^ Mann–Whitney U-test, ^2^ Kruskal–Wallis test. Significant values are given in bold.

**Table 3:** Descriptive data on aspects of caring in the two genders and three relationship subgroups (n = 360).

Variables	Total (n = 360)	Female (n = 229)	Male (n = 131)	*p*-Value ^1^	Spouse (n = 110)	Adult Child (n = 215)	Other Family Member (n = 35)	*p*-Value ^2^
Number of hours caregiving per week (n 338)				0.596				**0.000**
Mean (SD)	11.9 (11.8)	12.2 (12.5)	11.4 (10.7)		19.3 (16.7)	9.3 (7.6)	7.1 (6.3)	
Number of caregiving activities (n 357)								
Mean (SD)	5.5 (2.8)	6 (2.7)	5.2 (2.9)	0.114	6.5 (3.6)	5.2 (2.2)	4.6 (2.4)	**0.000**
	**n (%)**	**n (%)**	**n (%)**		**n (%)**	**n (%)**	**n (%)**	
Feeling responsible for the older person’s wellbeing (n = 355)				0.948				0.959
High degree	327 (91)	208 (92)	119 (92)		99 (92)	196 (92.5)	32 (91)	
Some degree	23 (7)	15 (6.5)	8 (6)		7 (6)	13 (6)	3 (9)	
Low degree	2 (1)	1 (0.5)	1 (1)		0 (0)	2 (1)	0 (0)	
Not at all	3 (1)	2 (1)	1 (1)		2 (2)	1 (0.5)	0 (0)	
Feeling responsible for the older person getting sufficient formal assistance (n = 355)				0.939				**0.001**
High degree	303 (85)	193 (85)	110 (85)		88 (82)	191 (90)	24 (68)	
Some degree	28 (8)	19 (9)	9 (7)		9 (8)	16 (7.5)	3 (9)	
Low degree	13 (4)	7 (3)	6 (5)		7 (6)	1 (0.5)	5 (14)	
Not at all	11 (3)	7 (3)	4 (3)		4 (4)	4 (2)	3 (9)	
Feeling powerless (n = 354)				**0.007**				**0.010**
Very often	63 (18)	51 (23)	12 (9)		26 (24)	35 (17)	2 (6)	
Often	148 (42)	91 (40)	57 (44)		40 (37)	94 (44)	14 (40)	
Seldom	112 (31)	66 (29)	46 (36)		35 (33)	68 (32)	9 (26)	
Never	31 (9)	17 (8)	14 (11)		6 (6)	15 (7)	10 (28)	
Feeling guilt (n = 354)				**0.034**				**0.029**
Very often	67 (19)	47 (21)	20 (16)		19 (18)	42 (20)	6 (17)	
Often	121 (34)	82 (36)	39 (30)		38 (35)	75 (35)	8 (23)	
Seldom	105 (30)	62 (28)	43 (33)		21 (20)	76 (36)	8 (23)	
Never	61 (17)	34 (15)	27 (21)		29 (27)	19 (9)	13 (37)	
Feeling of not helping enough (n = 354)				0.083				0.116
Very often	46 (13)	32 (14)	14 (11)		13 (12)	31 (15)	2 (6)	
Often	104 (29)	72 (32)	32 (25)		37 (35)	57 (27)	10 (28.5)	
Seldom	147 (42)	87 (39)	60 (46)		30 (28)	104 (49)	13 (37)	
Never	57 (16)	34 (15)	23 (18)		27 (25)	20 (9)	10 (28.5)	
Receive enough support from the health and social services (n = 360)				**0.020**				**0.041** ^3^
Always	23 (6)	12 (5)	11 (8)		8 (7)	10 (5)	5 (14)	
Often	27 (8)	14 (6)	13 (10)		11 (10)	13 (6)	3 (9)	
Sometimes	36 (10)	28 (12)	8 (6)		10 (9)	20 (9)	6 (17)	
Seldom/never	74 (21)	55 (24)	19 (15)		22 (20)	48 (22)	4 (11)	
Not currently	200 (55)	120 (53)	80 (61)		59 (54)	124 (58)	17 (49)	
Getting enough help as a supportive person? (n = 360)				0.631				**0.023** ^3^
Always	3 (1)	3 (1)	0 (0)		3 (3)	0 (0)	0 (0)	
Often	15 (4)	9 (4)	6 (5)		5 (5)	8 (4)	2 (6)	
Sometimes	27 (8)	21 (9)	6 (5)		13 (12)	10 (5)	4 (11)	
Seldom/never	105 (29)	72 (32)	33 (25)		28 (25)	68 (31)	9 (26)	
Not currently	210 (58)	124 (54)	86 (65)		61 (55)	129 (60)	20 (57)	

Note to Table 3: ^1^ Mann–Whitney U-test, ^2^ Kruskal–Wallis test. Significant values are given in bold, and ^3^ post hoc analysis was not performed due to the high proportion of “Not currently”.

**Table 4:** Logistic regression analysis concerning general and mental health in the total study group (n = 360), adjusted for gender and relationship.

Care Aspects	Poor General Health			Anxiety			Depression		
	OR	CI (95%)	*p*-Value	OR	CI (95%)	*p*-Value	OR	CI (95%)	*p*-Value
>6 h caregiving per week	2.31	1.31–4.08	**0.004**	1.59	0.89–2.85	0.116	2.59	1.46–4.62	**0.001**
>13 caregiving activities	1.20	0.69–2.09	0.523	1.17	0.68–2.01	0.579	1.07	0.62–1.85	0.801
Feeling responsible for the older person’s wellbeing	0.27	0.13–5.41	0.391	0.35	0.20–6.03	0.470	0.28	0.14–5.71	0.410
Feeling responsible for the older person getting sufficient formal assistance	0.16	0.38–0.69	**0.013**	0.81	0.19–3.49	0.775	1.39	0.25–7.70	0.710
Feeling powerless	2.63	1.39–4.97	**0.003**	6.95	3.72–12.99	**<0.001**	3.29	1.80–6.02	**<0.001**
Feeling guilty	1.11	0.50–2.46	0.780	1.05	0.47–2.36	0.905	1.73	0.81–3.73	0.159
Feeling of not helping enough	1.06	0.52–2.16	0.865	0.63	0.30–1.32	0.224	1.15	0.58–2.31	0.687

OR, odds ratio; CI, confidence interval. A *p* value ≤ 0.005 was considered significant. Significant values are given in bold. The two items with a high proportion of the answer “Not currently”, i.e., “Receive enough support from the health and social services” and “Receive enough help as a supportive person”, were excluded from the regression analyses.

## Data Availability

The datasets used and analysed during the study are available from the project leader (G.A.) upon written request and in accordance with ethical approval.

## Supplementary Materials

The following supporting information can be downloaded at: https://www.mdpi.com/article/10.3390/nursrep14020069/s1, Supplementary File S1: Data coding of the response alternatives for the logistic regression analyses.

## Public Involvement Statement

No public involvement in any aspect of this research.

## Guidelines and Standards Statement

This manuscript was drafted against the Strengthening the Reporting of Observational Studies in Epidemiology (STROBE) Statement: STROBE checklist for reporting cross-sectional studies.
