# Supplementary material for: Caring and Health of Close Family Members of Frail Older Persons Recently Discharged from Acute Hospital Care: A Comparative Cross-Sectional Study
Source: Nurs Rep. 2024 Apr 10;14(2):901–12. doi: 10.3390/nursrep14020069 (PMC11036298; doi:10.3390/nursrep14020069)
Supplement: Supplementary file 1 [file nursrep-14-00069-s001.zip › nursrep-2907461-supplementary.pdf]

Supplementary File S1. Data coding of the response alternatives for the logistic regression analyses

Variables	Response alternatives	Dichotomised
**DEPENDENT VARIABLES**		
**General health**	1 Very good<br>2 Rather good<br>3 Somewhat<br>4 Rather poor<br>5 Very poor	0 = good health (score 1 and 2),<br>1 = poor health (score 3-5)
**HAD-A (anxiety)**	Score 0-11	0 = no anxiety (score 0-6),<br>1 = anxiety (score ≥ 7)
**HAD-D (depression)**	Score 0-11	0 = no depression (score 0-6),<br>1 = depression (score ≥ 7)
**INDEPENDENT VARIABLES**		
**Number of caregiving activities**	13 different activities to choose from	Mean 5 was used to dichotomise the group<br>0 = ≤ 5 and 1 = ≥ 6
**Number of hours caregiving per week**	hours/week	Mean 11.9 was used to dichotomise the group<br>0 = ≤ 12 h/week and 1= ≥ 13 h/week
**Feeling responsible for the older person's wellbeing**	1 Not at all<br>2 Low degree<br>3 Some degree<br>4 High degree	0 = Not at all<br>1 = High degree, Some degree, and Low degree
**Feeling responsible for the older person's getting sufficient formal assistance**	Same as above	
**Feeling powerless**	1 Never<br>2 Seldom<br>3 Often<br>4 Very often	0 = Seldom and Never<br>1= Very often and Often
**Feeling guilty**	Same as above	
**Feeling of not helping enough**	Same as above	
